# IMPACTS OF PAYMENT REFORM AND COVID-19 ON POSTACUTE CARE ACCESS FOR OLDER ADULTS WITH DISABILITIES

**DOI:** 10.1093/geroni/igae098.2459

**Published:** 2024-12-31

**Authors:** Rachel Prusynski, Natalie Leland, Andrew Humbert, Arati Dahal, Cait Brown, Harsha Amaravadi, Tracy Mroz

**Affiliations:** University of Washington, Seattle, Washington, United States; University of Pittsburgh, Pittsburgh, Pennsylvania, United States; University of Washington, Seattle, Washington, United States; University of Washington, Seattle, Washington, United States; University of Washington, Seattle, Washington, United States; University of Washington, Seattle, Washington, United States; University of Washington, Seattle, Washington, United States

## Abstract

Recent Medicare payment reforms, the Patient-Driven Payment Model (PDPM) for skilled nursing facilities (SNFs) and the Patient-Driven Groupings Model (PDGM) for home health agencies (HHAs), aim to improve access to post-acute care for patients with complex care needs by focusing reimbursement on diagnosis and medical complexity. Caring for complex patients, such as those with disabilities, under previous systems may have been less profitable for providers, discouraging admissions. We examined whether these policies improved access to SNF and HHAs for beneficiaries with disabilities. We conducted an interrupted time series analysis of Medicare hospitalizations from 2018-2021 to identify changes in access to SNFs or HHAs. This period included reform implementation and the COVID-19 pandemic, which emerged shortly after reforms. We identified 7,734,908 hospitalizations for beneficiaries with a disability as the original reason for Medicare entitlement. Before reforms, 11.1% of beneficiaries with disabilities were discharged to SNFs and 13.1% to HHAs. PDPM was associated with no difference in HHA discharges but a 0.43-percentage point (pp) decline for SNF. PDGM was associated with increases in HHA (1.91pp) and SNF (0.37pp) discharges until the COVID-19 pandemic, when HHA discharges again increased, remaining high at an average of 18.4% after vaccination efforts. SNF discharges declined at the onset of the pandemic but increased to 14.3% of all hospital discharges after vaccination efforts. Results suggest PDPM worsened access to SNF for beneficiaries with disabilities, but SNF access rebounded after COVID-19 vaccinations. Alternatively, PDGM improved access to HHAs for beneficiaries with disabilities, which remained high during the pandemic.

